# Louping Ill Virus Genome Sequence Derived from the Spinal Cord of an Infected Lamb

**DOI:** 10.1128/genomeA.00454-13

**Published:** 2013-07-18

**Authors:** Denise A. Marston, Karen L. Mansfield, Rebecca Mearns, Richard J. Ellis, Anthony R. Fooks, Nicholas Johnson

**Affiliations:** Wildlife Zoonosis and Vector-Borne Diseases Research Group, Animal Health and Veterinary Laboratories Agency, Addlestone, Surrey, United Kingdoma; Animal Health and Veterinary Laboratories Agency–Penrith, Merrythought, Calthwaite, Penrith, United Kingdomb; Specialist Scientific Support Department, Animal Health and Veterinary Laboratories Agency, Addlestone, Surrey, United Kingdomc; University of Liverpool, Department of Clinical Infection, Microbiology and Immunology, Liverpool, United Kingdomd

## Abstract

Louping ill virus (LIV) is a zoonotic virus causing fatal encephalitis in young sheep and grouse. We have recovered the complete genome sequence from a spinal cord sample prepared from a lamb that was naturally infected with LIV. This is only the second LIV genome sequence reported and the first prepared from a clinical sample.

## GENOME ANNOUNCEMENT

The tick-borne *Flavivirus* genome consists of a positive-sense RNA, approximately 10,800 bp in length (1). This encodes a single polyprotein that is posttranslationally cleaved to form three structural proteins and seven nonstructural proteins (2). Louping ill virus (LIV) is genetically similar to tick-borne encephalitis virus (TBEV), and it has been proposed that they form a single species with four viral types (3). LIV is distinct from TBEV in that it is prevalent in the United Kingdom, in the absence of TBEV, and is virulent in sheep, causing fatal encephalitis. TBEV does not cause disease in sheep but is highly virulent in humans. A single LIV genome sequence is currently available in GenBank (that of LIV strain 369/T2; accession no. NC_001809). This was obtained from a virus isolated from a Scottish *Ixodes ricinus* tick in 1963 and was sequenced after a long passage history in the late 1990s (4). In contrast to LIV, TBEV has almost 50 complete genome sequences available. The generation of additional genomic data for LIV will assist further investigations into the properties of this virus.

Total RNA was extracted using the TRIzol method from a spinal cord sample prepared from a young sheep that died suddenly near Penrith, England, in 2009. LIV infection in the sheep was confirmed independently by immunohistochemistry and detection of flavivirus by reverse transcription-PCR. RNA for whole-genome sequencing using pyrosequencing was prepared as previously described (5). Briefly, RNA was depleted of host genomic DNA using RNase-free DNase (Qiagen), and host rRNA was depleted using Terminator 5′-phosphate-dependent exonuclease (Epicentre Biotechnologies). Depleted RNA was fragmented, and a random-primed cDNA library was prepared and sequenced with the Roche 454 GS FLX system. Sequence data were assembled in the GS mapping assembly software (Roche) using the currently available LIV sequence (accession no. NC_001809). This approach recovered a partial genome sequence with only 77 viral reads (0.1% of total reads), reflecting the relatively small proportion of virus genome in relation to host nucleic acids. The remaining sequence was obtained using directed PCR and Sanger sequencing of genomic gaps. The contigs from both approaches were assembled using SeqMan (DNAStar).

The LIV Penrith genome is 10,875 nucleotides long with 95.6% identity to the existing LIV genome. The polyprotein-coding sequence is 10,245 nucleotides and contains 3,415 codons. The 5′ and 3′ untranslated regions (UTR) are 131 and 499 nucleotides, respectively. A comparison with the existing LIV genome sequence (that of strain 369/T2) suggests conservation within the polyprotein region, which is the same length and is 97.5% identical at the amino acid level. Both the 5′ UTR and the 3′ UTR of LIV 369/T2 are 2 nucleotides longer than those of LIV Penrith. This is the first complete LIV genome sequence from England and the first LIV sequence from primary diagnostic material. Additional sequences from LIV isolates and samples from across its geographical range should enhance our understanding of this neglected virus.

### Nucleotide sequence accession number.

The complete genome sequence of LIV Penrith has been deposited in GenBank under the accession no. KF056331.
